# Physical Exercise for Healthy Older Adults and Those with Frailty: What Exercise Is Best and Is There a Difference? A Systematic Review and Meta-Analyses

**DOI:** 10.1155/2024/5639004

**Published:** 2024-07-05

**Authors:** Samaher Alowaydhah, Ishanka Weerasekara, Sarah Walmsley, Jodie Marquez

**Affiliations:** ^1^College of Health, Medicine and Wellbeing, The University of Newcastle, Newcastle, Australia; ^2^College of Applied Medical Science, Jouf University, Sakakah, Saudi Arabia; ^3^Faculty of Health and Social Sciences, Western Norway University of Applied Sciences, Bergen 5063, Norway; ^4^School of Allied Health Science and Practice, Faculty of Health and Medical Sciences, The University of Adelaide, Adelaide, SA 5005, Australia; ^5^Hunter Medical Research Institute, New Lambton, Australia

## Abstract

**Methods:**

All English studies published after 1989 with a controlled design, investigating *PE* *in* *adults* 65 years and over were considered if the study design compared PE to a nonexercise control group. Health-related outcomes included physical, cognitive, and psychological function. Studies that investigated cardiorespiratory disease and used designs like systematic review were excluded. *Results and Discussion*. Altogether, 57 studies were included of which 38 had data that were useable for meta-analysis. In the healthy aged, a significant benefit of multicomponent exercises (*p*=0.006, SMD = 1.40, CI = 0.41, 2.40) and tai chi (*p*=0.01, MD = 0.51, CI = 0.12, 0.91) on physical function was revealed, while strength exercise benefitted cognitive function (*p*=0.04, SMD = 0.86, CI = 0.03, 1.68). In frail older adults, there was a significant benefit of multicomponent exercises on physical function (*p* < 0.0001, SMD = −10.85, CI = 5.66, 16.04) and mental health (*p*=0.0002, SMD = −0.39, CI=-0.18, 0.59). Strength exercise had a significant benefit on activity of daily living (ADL) (*p* < 0.0003, SMD = 15.78, CI = 7.28, 24.28).

**Conclusion:**

The substantial disparity of research in the field of exercise in older adults renders synthesis of the evidence problematic. However, it appears that multicomponent exercise is the most suitable approach for both healthy and frail older adults although the benefit may be reflected in different health outcomes.

## 1. Introduction

In 2019, the number of people aged 65 and over globally was estimated to be 703 million [1]. By 2050, this number is expected to be 1.5 billion, and those aged 80 or over will represent 426 million people [1, 2]. As increased longevity is associated with increased chronic illness and morbidity, hospitalization falls, and increased demand for support and care [3, 4], many older adults are classified as being frail. Although a clear definition has not been established for the term frailty, it is commonly used to define people who are characterized by slow gait speed, weak grip strength, exhaustion, reduced energy expenditure, and weight loss [3, 5]. Some authors also include mental health as an important domain that should be considered when defining frailty [6]. The global incidence of frailty among community-dwelling older adult people is estimated to be 43.4% [7].

The maintenance of health status and functioning with aging has been identified as a critical issue globally critical issue, and as a result, a number of countries have developed programs to facilitate healthy aging [8, 9]. This involves consideration of the complex relationship between individual attributes and behaviours, as well as social, economic, and environmental contexts [10]. Central to this is the need for physical exercise (PE) [11].

There is an extensive body of research investigating the effects of PE across the lifespan; however, the research is disparate regarding types of exercise, exercise settings (independent, supervised, or group), dosage of exercise including frequency and intensity, and specific recommendations for subgroups of older adults such as those who are frail.

The aim of this systematic review and meta-analysis was therefore to synthesize the current evidence regarding the prescription of exercise in healthy, as well as frail, older people in an attempt to understand if the response to exercise is the same and if exercise prescription needs to be further tailored to meet the needs of the frail subgroup. To ensure relevance, articles were restricted to those published within the last 30 years and prior to the onset of COVID-19, considering the potential changes in exercise delivery, recruitment, and engagement during this time [12].

Specifically, the objectives of the current systematic review were to establish which types of exercise are most beneficial and whether this differs between those who are healthy and those who are frail.

## 2. Methods

This review forms part of an overarching review that seeks to establish the benefits of various types of exercise for older adult subgroups. In addition to healthy and frail older adults, this work includes subgroups of those with prevalent conditions in older people such as cognitive, orthopedic, and neurological diseases. These findings will be reported elsewhere. This review followed the Preferred Reporting Items for Systematic Reviews and Meta-Analyses (PRISMA) statement [13]. The protocol was registered under the registration number CRD42020173465 in an international prospective register of systematic reviews (PROSPERO) on 28 April 2020.

### 2.1. Search Strategy

The principal electronic search of Medline, Cochrane, Embase, CINAHL, and Scopus was conducted in March 2020. The search strategy was conducted using key terms related to “exercise” and “geriatric.” No methodological filter was used for the study design to maximize detection; however, a time limit for studies published after 1989 was applied to ensure alignment with contemporary exercise implementation practices. For the same reasons, the search did not include research onwards the onset of the COVID pandemic, as many programs were necessarily modified and therefore may not have been heterogenous for meta-analysis with regard to recruitment, implementation, adherence, and outcomes. The search strategy used for MEDLINE is provided in (Sapp1).

### 2.2. Criteria Used for Selecting Studies

Studies were eligible for inclusion if they met the following criteria: (a) included human adults ≥65 years or older on average and (b) used a PE intervention, as defined below, compared with a control condition. Excluded studies (a) were published prior to the year 1990, (b) were published in languages other than English, (c) investigated cardiac, respiratory, and postsurgery populations, and (d) used study designs such as protocols, systematic reviews, editorials, commentaries, reports, conference abstracts, case studies, and case series.

In the case where there was more than one publication for the same study, for example, an additional publication including longitudinal data, the most recent study only was included to avoid duplication of participants. Both studies were included if different outcomes were reported in each of the publications. The four-stage PICO format (Population, Intervention, Comparison, and Outcome) was used as clarified as follows.

### 2.3. Population

The population of interest was any adults over the age of 65 who were defined by the original authors as either healthy or frail. In the instance where the study population was of mixed age, studies were included if the average age of the study population was greater than 65 years. Studies, where the populations of interest were those with cardiac or respiratory diseases, recent fractures, or patients' postsurgery, were excluded from the review as these subgroups require specific exercise considerations.

### 2.4. Intervention

We selected studies if they investigated any form of PE intervention as described by the definition of a structured, arranged, and repeated physical activity aimed at enhancing or maintaining one or more health-related factors. This included individual exercise or group settings. Further, physical exercise interventions that were combined with cognitive interventions or dietary interventions were also excluded if an independent exercise comparator group was not included.

### 2.5. Comparison

Only studies with a control condition were considered. This included usual care (e.g., occupational therapy or no care), different types of intervention (e.g., cognitive behavioural therapy), or wait-list control. Studies that compared different types of exercise interventions and did not have a nonexercise control condition were excluded because the act of comparing two exercise groups does not provide us with the means to determine the benefit of exercise.

### 2.6. Outcome Measures

We were interested in the benefits of PE across a broad spectrum of health-related outcomes ranging from impairment measures to quality of life. Studies that only included physiological measures such as brain activity or cellular mediated responses, or frequency of falls, were excluded. For ease of description, we grouped outcomes under broad terms where appropriate, for example, “physical health and function” included and measure of performance of physical tasks such as gait, balance, and strength, and “mental health” included depression and anxiety. Where a study included more than one measure of the same outcome, e.g., both the HADS and Kessler scale to assess mood, the data from the tool with the greatest frequency of use in the selected articles were included in the analysis. For cognition, in the absence of a common tool, the MMSE was prioritised, then the Montreal Cognitive Assessment (MoCA), then the tool that evaluated memory, followed by the tool that was identified by the authors as the primary tool of interest.

### 2.7. Study Selection Process

Search data were exported to Endnote reference software, and then Covidence software (Covidence systematic review software, Veritas Health Innovation, Melbourne, Australia) for screening. After removing duplicates, title/abstracts were independently reviewed by two reviewers (SA, IW) and then full texts were retrieved and screened for eligibility (SA, IW). Disagreements at both levels were resolved by a third reviewer (JM).

### 2.8. Screening and Data Extraction

Data for assessing study quality, descriptive data, and quantitative data for the calculation of effect size were extracted and recorded by one researcher (SA). A second researcher (IW) cross-checked the extracted data for accuracy. Discrepancies were resolved following discussion with the research team. The methodological quality of the individual-included studies was determined using the Physiotherapy Evidence Database (PEDro) scale [14].

### 2.9. Data Analysis

A descriptive analysis of the extracted data was undertaken, and the findings were tabulated and graphically presented to answer the research objectives. Where sufficient quantitative data were available, it was pooled and meta-analysis was performed. The Cochrane statistical package, Review Manager 5 (RevMan 5.4), was used for statistical analyses. A fixed effects model was applied if heterogeneity was low as assessed by the *I*^2^ index. If *I*^2^ was >30%, a random-effects model was used to incorporate intertrial heterogeneity [15]. For continuous data, mean difference (MD) was used if the same tool was used. Standard mean difference (SMD) was used when different tools were used to assess the same outcome employing 95% confidence intervals (CIs) to evaluate the effect size. In the instance where a decrease in the score of the tool indicated an improvement (e.g., the timed-up and go test), the inverse of the change score was used so that positive change scores consistently represented performance improvements. This review followed the general practice of interpretation for small, medium, and large effect sizes (0.2, small effect; 0.5, medium effect; 0.8, large effect) [15]. Where there were insufficient data for meta-analyses, or if there were single studies exercise type [16–34], the results were reported narratively.

## 3. Results

**Figure 1:**
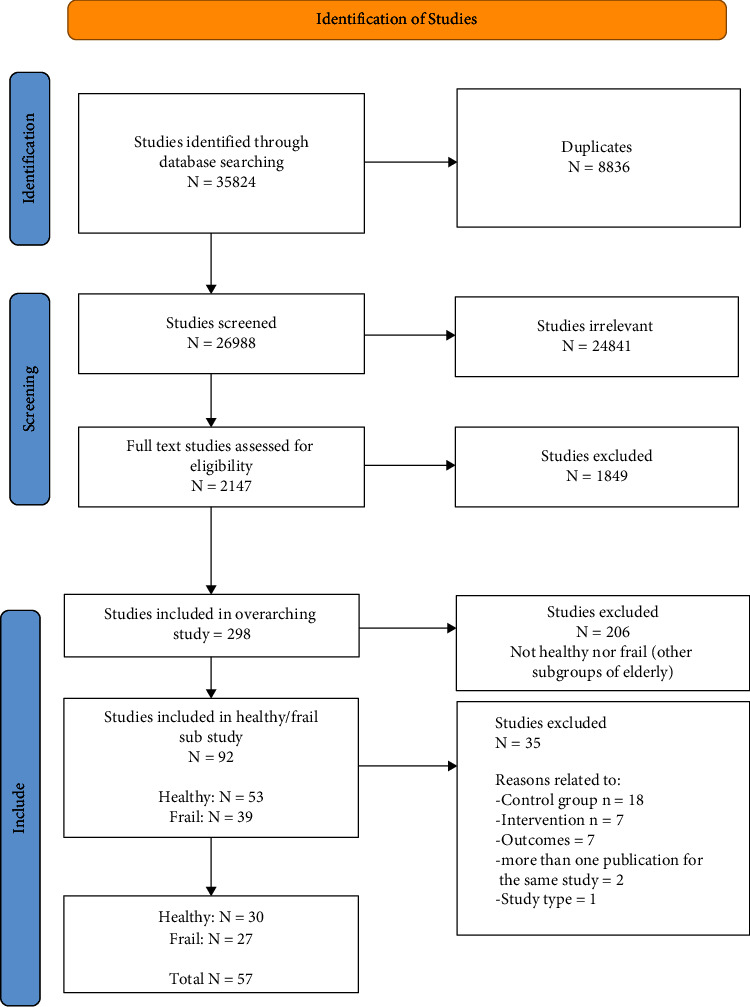
Flow of studies through the review.

The database search for the overarching study identified 35824 articles. Following the removal of duplicates and abstracts, 2,147 articles were screened for eligibility, of which 92 articles were reviewed for relevance to this substudy. Thirty-five of these articles were excluded with the main reason being for lack of adequate control condition. In total, 57 studies were included; of these, 38 had data available for meta-analysis.  Figure 1 describes the flow of studies through the review.

### 3.1. Quality of the Included Studies

The methodological quality of the included 57 studies ranged from low to high with a mean PEDro score of 5.5 out of 10 (SD 1.3, range 3–8). This was relatively consistent between the two samples with the healthy older adult studies having a slightly lower quality on average (mean = 5.4, SD = 1.3) than the studies with frail participants (mean = 5.6, SD = 1.3). The majority of studies used randomization (94.7%); however, only 24.6% reported concealed allocation. The worst performing item on the quality assessment was blinding, whereby only two studies (3.5%) reported participant blinding, and no studies reported blinding of the therapists (0%). Sapp 2 details the quality assessment scores.

### 3.2. Summary of Included Studies

Thirty studies investigated the effects of exercise in 2,370 healthy older adults. The mean age of participants was 74.6 years. The majority of studies were conducted with community-dwelling participants, with one study recruiting participants from a retirement village [35] and another from an outpatient clinic [36]. Studies occurred across a range of countries, with the highest frequency occurring in Japan [23, 37–41], and the USA [17, 22, 42–44]. The majority, of 15 studies, implemented multicomponent exercise programs, using various combinations of strength, flexibility/stretching, balance, coordination, and aerobic exercise [22, 35, 38–50]. The remaining studies investigated one specific exercise type, including strength training [18, 42, 46, 51–54], tai chi [37, 55], aerobic or endurance exercise [16, 21, 23], balance [42], inspiratory muscle training [19], yoga [17], dancing [36, 56], flexibility [47], and aquatic exercise [20]. The duration of PE varied from six weeks [17] to 52 weeks [21, 37, 41] with 60 minutes being the most commonly used across the studies three times per week. Most studies delivered exercise interventions as group classes (19 studies), while nine used individual supervision [16, 20, 23, 43, 47, 48, 52, 54, 55] or independent exercises [19, 50].  Table 1 provides a full description of the included studies.

Twenty-seven studies examined the effects of exercise on 3,782 frail older adults. The average age of participants was 80.3 years. Eight studies recruited participants from community settings [26, 28, 30–32, 34, 57, 58], 14 involved participants from residential care facilities [24, 25, 27, 33, 59–68], four from hospital and outpatient clinics, and one was not specified [69]. The studies were conducted across the globe with the most common populations being in the USA [30, 34, 57, 62, 69] and Japan [27, 61, 68]. The majority of studies, 14 studies, provided multicomponent PE programs [28–31, 59, 60, 63, 65, 67–72]. Five studies investigated tai chi [25, 27, 34, 57, 61], and the remaining reviewed aerobic exercise [24, 31, 62, 64], balance [26, 33, 34], and strength exercise [31, 32, 58, 63, 66]. The duration of the intervention varied widely across the studies from four weeks [33] to 104 weeks [30]. The most prevalent session duration was 45 minutes [24–26, 29, 31, 66, 67, 70], with a range from 15 minutes [34, 64] to 90 minutes [57, 72]. See Table 2 for a full description of the included studies.

#### 3.2.1. Multicomponent Exercise

**Figure 2:**
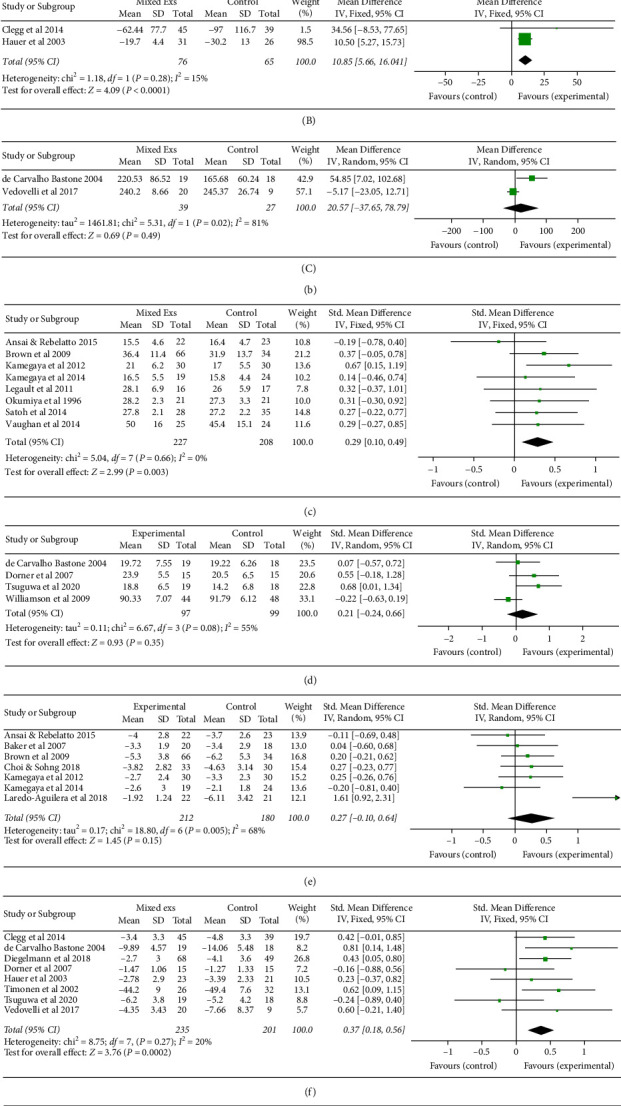
The effect of multicomponent exercises on specific outcomes. (A) Physical health and function of healthy subjects. (B) Physical performance as measured by TUG of the healthy subject. (C) Physical performance as measured by SPPB of healthy subjects. (A) Physical health and function of frail subjects. (B) Physical performance as measured by TUG of frail subjects. (C) Physical performance as measured by 6MWT of frail subjects. (c) Cognitive function of healthy subjects. (d) Cognitive function of frail subjects. (e) Depression of healthy subjects. (f) Depression of frail subjects. (g) Quality of life of healthy subjects. (h) Activity of daily living of frail subjects. (i) Mobility of healthy subjects. (j) Mobility of frail subjects.

*(1) Physical Function*. PE programs that involved a combination of exercise types improved at least one measure of physical function in seven out of ten studies [22, 35, 38, 42, 45, 48, 50] with healthy participants and six out of nine studies [28, 31, 59, 60, 68, 70] with frail participants. When data were pooled from eight studies with 532 healthy participants, there were no significant effects of multicomponent exercises on physical performance (*p*=0.22, SMD = 0.29, CI = −0.18, 0.76) [35, 38–40, 45, 46, 49, 51] (Figure 2(A)). Physical performance measured by Timed Up and Go (TUG) and Short Physical Performance Battery score (SPPB) was analyzed further separately given that there were adequate data. There was a significant effect of multicomponent exercises on TUG when data from 245 healthy participants were pooled (*p*=0.006, MD = 1.40, CI = 0.41, 2.40) [38–40, 48, 50] (Figure 2(B)). There was no significant effect of multicomponent exercises on SPPB when data from 275 healthy participants were pooled together (*p*=0.30, MD = 0.19, CI = −0.17, 0.54) [35, 44, 50] (Figure 2(C)).

Data from five studies with 237 frail participants were pooled together for meta-analysis. No significant effect of multicomponent exercises on physical performance was observed (*p*=0.06, SMD = 0.44, CI = −0.01, 0.88) [59, 60, 65, 70, 71] (Figure 2(A)). Functional performance measured by TUG and 6MWT was separately analyzed, given that there were adequate data for subanalysis. There was a significant effect of multicomponent exercises on TUG when data from 141 frail participants were pooled (*p* < 0.0001, MD = −10.85, CI = 5.66, 16.04) [70, 71] (Figure 2(B)). Conversely, there was no significant effect of multicomponent exercises on 6MWT when data from 66 frail participants were pooled (*p*=0.49, MD = 20.57, CI = −37.65, 78.79) [59, 65] (Figure 2(C)).

*(2) Cognitive Function*. The effect of multicomponent exercise on cognitive function was assessed in eight studies, of which five studies reported significant benefit compared to control in healthy older adults [39–41, 47, 48] and four of the eight studies assessed this outcome in the frail [28, 29, 65, 68]. Regarding healthy older adults, data were available for meta-analysis from eight studies [38–41, 43, 46–48]. Pooled data from 435 healthy participants revealed a significant effect of multicomponent exercises on cognitive function in older adults (*p*=0.003, SMD = 0.29, CI = 0.10, 0.49) (Figure 2(c)).

Data were available for meta-analysis in four studies with 196 frail participants. There was no significant effect of multicomponent exercises on cognitive function (*p*=0.35, SMD = 0.21, CI = −0.24, 0.66) [59, 60, 68, 69] (Figure 2(d)).

*(3) Mental Health and Wellbeing*. Nine studies investigated the benefits of multicomponent exercise programs on mental health in healthy older adults. Of these, benefits were identified in four studies [35, 45, 47, 49]. Eight studies assessed the effects of multicomponent exercise on depression with 436 frail participants. Seven studies with a total of 392 healthy participants had data available for meta-analysis. No significant improvement in depression, compared to control, was found (*p*=0.15, SMD = −0.27, CI = −0.10, 0.64) [35, 39, 40, 45–47, 49] (Figure 2(e)).

When data from the eight studies with 436 frail older adults were combined, a significant benefit was revealed (*p*=0.0002, SMD = −0.37, CI = 0.18, 0.56) [59, 60, 65, 67, 68, 70–72] (Figure 2(f)).

One study assessed anxiety using the Beck Anxiety Inventory (BAI), no significant effect of multicomponent exercises on anxiety for frail older adults was observed (*p* >  0.05) [65].

*(4) Quality of Life*. Quality of life was investigated in three studies with healthy participants, and significant benefits were reported in two of these studies [22, 39]. Meta-analysis of two studies with 103 healthy participants showed no significant effect of multicomponent exercise on quality of life (QOL) (*p*=0.58, SMD = 0.50, CI = −1.28, 2.28) [39, 40] (Figure 2(g)).

One study investigated the effects of a multicomponent of frail older adults on QoL [28]. This study demonstrated that 60-minute exercise sessions over 12 weeks resulted in significant improvement in quality of life as measured on the Quality-of-Life Systemic Inventory Questionnaire (*p*=0.05).

*(5) Activities of Daily Living*. No studies with healthy older adults evaluated activities of daily living (ADL) as an outcome of interest. Four studies and 208 frail participants were pooled to reveal no significant effect of multicomponent exercises on ADL (*p*=0.43, MD = 0.61, CI = −0.92, 2.15) [60, 68, 70, 71] (Figure 2(h)).

*(6) Mobility*. Four studies with a total 280 healthy older adults pooled together [35, 39, 42, 44]. No significant improvement was found (*p*=0.07, MD = −0.23, CI = −0.47, 0.02) (Figure 2(i)).

Data for mobility were pooled together from two studies with 87 frail participants. There was no significant effect of multicomponent exercises on the mobility of the frail older adult (*p*=0.35, SMD = 0.53, CI = −0.57, 1.62) [60–68, 70, 71] (Figure 2(j)).

#### 3.2.2. Tai Chi Exercise

*(1) Physical Function*. Two studies investigated the effects of tai chi interventions [37, 55]. When 129 healthy participants pooled together, there was a significant effect of tai chi on physical function (*p*=0.01, MD = 0.51, CI = 0.12, 0.91) [37, 55] (Sfig 3A).

Three studies investigated the effect of tai chi on physical performance outcomes of frail older adults with all three reporting improvement compared to the control [25, 34, 57] [25]. There were no common data available for meta-analysis.

*(2) Cognitive Function*. Cognitive function was investigated in one study and 40 healthy participants, whereby significant benefit on healthy older adults was revealed on the Chinese Dementia Rating Scale (*p*=0.04) but not the MMSE (*p*=0.36) [55].

No studies with frail older adults measure the effect of tai chi on cognitive function.

*(3) Mental Health and Wellbeing*. No studies with healthy older adults evaluated mental health as an outcome. Data from two studies with 138 frail participants were pooled [27, 57]. No significant effect of tai chi on depression was found (*p*=0.59, SMD = −0.31, CI = −0.80, 1.42) (Sfig 3B).

*(4) Quality of Life*. QoL was not evaluated in any included studies with healthy older adults. One study investigated the effects of tai chi on QoL of frail older adults [61] and reported that 40 mins of seated tai chi across 26 weeks resulted in significant improvement in QoL as measured by the World Health Organization Quality of Life (WHOQoL) (*p*=0.03).

*(5) Activity of Daily Living*. No studies with healthy older adults evaluated ADL as an outcome. Two studies measured the effect of tai chi on ADL in frail older adults [25, 34] with one study reporting a significant benefit [25]. It was not possible to pool the data.

*(6) Mobility*. There was one study that measured the effect of 60 minutes of tai chi for 52 weeks on mobility for healthy older adults, and there was a significant improvement (*p*=0.015) [37]. One study evaluated the mobility as an outcome for frail older adults. Significant improvement was reported for white people by using ≥0.97 m/s or <0.97 m/s tool [57].

#### 3.2.3. Strengthening Exercises

*(1) Physical Function*. Strength training was beneficial for physical function in all four studies with healthy adults that evaluated this outcome [42, 51–53]. Data were pooled from two studies with 85 healthy participants [51, 52]. Both intervention groups (high-speed and low-speed training) of Yoon et al. were considered in the meta-analysis [51]. There was no significant improvement in strength training demonstrated (*p*=0.14, MD = 0.80, CI = −0.26, 1.86) (Sfig 4A). Four studies evaluated the benefit of strength training on physical function in frail older adults [31, 58, 63, 66]. All four reported benefits of strength training compared to control. No data were available for meta-analysis.

*(2) Cognitive Function*. Improvements in cognitive function were found in four studies evaluating this outcome in healthy participants [18, 46, 51, 53], and four included studies with frail participants [32, 58, 63, 66]. Three studies had data available from 129 healthy participants for meta-analysis [46, 51, 53]. Both intervention groups of Yoon et al. were included [51]. A significant effect of strengthening exercises on the cognitive function of older adults was revealed (*p*=0.04, SMD = 0.86, CI = 0.03, 1.68) (Sfig 4B).

For studies of frail adults, data were available for meta-analysis from three studies with 120 participants. No significant improvement was observed (*p*=0.14, SMD = 0.99, CI = −0.34, 2.31) [58, 63, 66] (Sfig 4C).

*(3) Mental Health and Wellbeing*. The data from two studies, with 179 healthy participants, were pooled for meta-analysis [46, 54]. The three intervention groups (resistance 1, 2, and 3) of Kekalainen 2018 were included. No significant benefit of PE was revealed (*p*=0.32, MD = 0.47, CI = −0.45, 1.39) (Sfig 4D). No studies with frail older adults evaluated mental health as an outcome.

*(4) Quality of Life*. Three studies evaluated the effect of strengthening exercise on QOL for healthy older adults with two studies reporting significant benefits [18, 54]. No data were available for meta-analysis. One study evaluated the effect of strengthening exercise on the quality of life of frail older adults. Following 12 weeks of resistance training for 60 minutes, twice a week, a significant benefit was reported (*p*  <  0.001) [63].

*(5) Activities of Daily Living*. No studies with healthy older adults evaluated ADL as an outcome. ADL was assessed using the Barthel Index (BI) in two studies with frail older adults [63, 66]. When the data for these 77 participants were pooled, a significant benefit for strength training was demonstrated (*p*=0.0003, MD = 15.78, CI = 7.28, 24.28) (Sfig 4E).

*(6) Mobility*. Two studies measured the effect of strength on the mobility of healthy older adults [42, 52]. When the studies were pooled together, a nonsignificant improvement was demonstrated (*p*=0.56, MD = −0.25, CI = −1.08, 0.59) (Sfig 4F).

One study assessed the effect of strength on the mobility of frail older adults and demonstrated a significant improvement (*p*=0.027) [58].

#### 3.2.4. Aerobic Exercises

*(1) Physical Function*. In healthy older adults, aerobic exercise did not lead to a significant benefit in physical function in the one study that evaluated this outcome using the Physical Health and Function test (*p*=0.13) [23]. In frail older adults, aerobic training was beneficial for physical function in two out of three studies [31, 62]. Data from two studies with 70 participants were pooled together for meta-analysis where the TUG was used to measure physical performance. No significant effect of aerobic exercises on physical performance was found (*p*=0.36, MD = 2.25, CI = −2.52, 7.02) [62, 64] (Sfig 5).

*(2) Cognitive Function*. With regard to cognition, in healthy older adults, two studies evaluated this outcome and reported significant benefits compared to the control [21, 23]. There were insufficient data for meta-analysis. Conversely, in the frail, no significant benefit was revealed in cognitive function in the one study that assessed this outcome [64].

*(3) Mental Health.* One study of healthy older adults evaluated mental health following aerobic exercise programs and reported significant benefits as measured with the BDI (*p*=0.01) [16]. Two studies evaluated the effect of aerobic exercise on mental health in frail older adults, with neither reporting significant benefit [24, 62].

*(4) Quality of Life*. There was no reported benefit of aerobic exercise on QoL in the one included study with healthy participants [23] (*p*=0.42) nor the study with frail older adults [62].

*(5) Mobility*. No study measured the effect of aerobic exercise on the mobility of healthy older adults. There was one study that evaluated mobility as an outcome of frail older adults; a nonsignificant improvement was reported [62]. No data are available.

#### 3.2.5. Dancing

Two studies investigated the effects of dancing on physical function in healthy older adults [36, 56]. When the data were pooled from 91 healthy participants, no significant effect was revealed (*p*=0.94 MD = 4.95, CI = −135.34, 145.25) (Sfig 6). One study reported the benefit of dancing on cognitive function as measured on the MoCA (*p*=0.003) and in mental health as measured on the GDS (*p*=0.01) [56]. Another showed a benefit in QoL for healthy older adults as measured on the 36-Item Short Form Survey (SF36) (*p*  <  0.05) [36]. No studies investigated the effects of dancing in frail older participants.

#### 3.2.6. Balance Exercise

One study found that balance exercises were beneficial for the physical health and function of healthy older adults (*p*  <  0.05) [42].

For frail older adults, balance training improved physical health and function outcomes in the three studies which evaluated this [26, 33, 34]. These studies also assessed the effect of balance on mental health [26, 33, 34], and none found a significant benefit. No included studies evaluated the effect of balance exercise on cognitive function.

#### 3.2.7. Single Study Exercise Types

Additional types of exercise were investigated in single studies to identify the effect of PE on healthy older adults. These were yoga, inspiratory muscle training, aquatic therapy, and flexibility. Bonura et al. 2014 concluded that six weeks of yoga resulted in improved anger control/mood than both routine exercise and control participants (Cohen's *d* = 0.89 for yoga versus exercise and 0.90 for yoga versus control [17]). Ferraro et al. 2019 used a program of twice-daily inspiratory muscle training over eight weeks to demonstrate significant improvements in functional performance as measured by the TUG compared to the control group (*p*=0.03) [19]. One study investigated supervised, individual aquatic exercises provided three times per week over 12 weeks. There were statistically significant improvements in depression (*p*=0.03) and fatigue (*p*=0.01) following the intervention, compared to the control [20]. Brown et al. 2009 reported that one hour twice a week of flexibility and relaxation techniques had significant effects on mental health measured by GDS (*p* < 0.01) [47]. There were no studies with frail participants that investigated these types of PE.  Table 3 describes the results of each study type and their outcomes. A visual representation of all meta-analyses conducted and the outcomes are presented in Sfig 7.

## 4. Discussion

This is the first systematic review and meta-analysis to comprehensively synthesize the current evidence regarding the prescription of exercise in both healthy and frail older people. It provides an understanding of the types of exercise programs which are most effective in these groups which may assist clinicians in the selection of appropriate interventions. The positive impacts of physical activity on various facets of health are widely recognized and accepted by all adults [73]. Health organizations worldwide have formulated guidelines outlining exercise recommendations, encompassing specific types of exercises, and suggesting intensity levels for engaging in these activities [74]. While some attention is given to healthy older adults, a considerable portion faces the challenge of frailty, which can hinder their ability to remain physically active. Therefore, it is important to tailor guidelines to maximize compliance and benefit and to provide overall better QoL with a less economic burden to communities in this subgroup. This systematic review has integrated the research findings from these two subgroups of the population to offer valuable insights into the best types of exercise that are associated with the greatest benefits in specific health-related outcomes and the difference between them.

Multicomponent exercise programs are the most widely investigated, and the evidence suggests that this form of exercise is beneficial for improving physical function in both healthy and frail older adults. In the healthy, it may have the added benefit of improving cognitive function, whereas in the frail, it may contribute to improved mood. Tai chi may also improve physical performance in the healthy aged; however, this benefit was not demonstrated in the frail population. Strengthening exercise programs may lead to improvements in cognition in healthy older adults and ADL performance in the frail. Drawing conclusions about other forms of exercise and other health-related outcomes is challenging due to the limited and heterogenous literature and inconsistent findings.

Our findings align with previous reviews exploring the advantages of exercise in healthy older adults [75]. Plummer et al. 2016 focused on the effects of PE on dual-task performance while walking among healthy older adults and revealed a notable increase in single-task gait speed [75]. This improved physical performance which was also observed in our review following multicomponent exercises and tai chi.

Several systematic reviews have specifically focused on the benefits of exercise in frail older adults with reference to specific outcomes [76–78]. Cadore et al. 2013 evaluated the effect of exercise on functional capacity in frail older adults, concluding that multicomponent interventions appear to be the most effective approach for improving these outcomes [76]. As well as concurring with our analysis, this may have implications for reducing fall-related morbidity, the decline in independence, and subsequent economic sequelae. Some previous authors have also evaluated the effects of physical exercise on physical function in addition to quality of life and activities of daily living [77, 78].

This study has a number of notable strengths as well as some limitations. Previous reviews have focused on singular exercise types or restricted outcomes which may limit their clinical application. This is the first systematic review to collate evidence, allowing comparisons across all forms of exercise and all available health-related outcomes in both healthy and frail older populations. We restricted inclusion to controlled trials with primarily good to excellent quality to minimize bias and allow for between-group comparisons. However, the review is constrained by the incongruity of the available evidence. Meta-analysis was limited by unusable data, a lack of studies investigating certain types of exercise such as yoga, and inconsistent use of outcome measures across studies. Where data were not available for meta-analysis, the findings were reported narratively. Given that the majority of studies were not powered (20 of the 57 included studies were powered) and failed to correct for multiple comparisons, caution must be taken in interpreting these findings as they are prone to type I and type II errors. Similarly, despite pooling the data, several of the meta-analyses contain samples with less than 90 participants; therefore, we are limited in the conclusion that can be drawn from these analyses. In some instances, the outcome tools used to evaluate change have not been validated and may not be responsive to change in this population, and care must be taken when interpreting these results. These tools include the Loss of Balance test, Sensory Organization test, muscle strength tests, Weschler Scale, COWAT, Satisfaction in Daily Life, and the Quality-of-Life Systemic Inventory. Further, some studies included subanalysis of their sample according to the presence or severity of symptoms such as the degree of cognitive impairment. It was beyond the scope of this review to incorporate such subgroup findings into our analysis. We relied on the definition and categorization of the sample as being either “healthy” or “frail” as provided by the original authors. However, the definition of frailty is not consistently reported nor uniformly applied, potentially leading to variations in how frailty was defined across the studies. Moreover, our review is restricted to studies published prior to 2020, due to the effects of COVID which may have impacted traditional exercise delivery. This may have resulted in missing out on some relevant studies.

In the future, it will be essential for researchers to adopt standardized approaches when it comes to exercise type, dosage, and the collection of outcomes to enable meaningful conclusions to be drawn. In some instances, it may be that the dosage of the intervention or the mode of the delivery was not targeted to achieve the measured outcomes' given current evidence. When considering all of the outcomes reported in this SR, there were a total of 30 reports of nonsignificant effects of these, and six studies did not report exercise adherence (37, 56, 54, 65, 70, 72) and a further three reported less than 80% adherence (40, 35, 59). Therefore, for these studies, it is not known whether failure to respond was due to inadequate adherence to the exercise and consequently insufficient dosage of the intervention. A further consideration is that our study included study samples that were predominantly female and despite this being representative of the older population, it may limit the generalizability to male populations. Lastly, this review may have overlooked relevant studies published in other languages because the searches were limited to English.

Despite the reported importance of physical exercise [79], there is a dearth of comprehensive, methodologically sound research investigating its effects on older adults. This deficit limits both clinical application as well as evidence-based policy direction. Essentially, our study demonstrates that physical exercise has a positive impact on both healthy and frail older adults in several domains. However, the specific benefits may vary depending on the type of exercise prescribed and the participants' health. As a result, we strongly urge clinicians to consider the particular population and the desired outcome when prescribing exercise in these populations to achieve the most relevant benefits. Further, we propose that our findings may assist in informing evidence-based guidelines and policies that will reduce the economic and social burden associated with inactivity in the older population.

## Figures and Tables

**Table 1:** Description of included studies with healthy older adults.

Author (year)	Sample size Female percentage (%)	Source of participants Age criteria (mean, range ∧ )	Intervention	Mode of delivery	Dose	Comparison	Duration (weeks)	Follow-up (months)	Main outcomes: tools	Other outcomes: tools	Main result
Ansai (2015)	69 powered 68	Community ≥80 years (82.4)	(1) Multicomponent (2) Resistance	Supervised Supervised	1 hour 3x/week	Usual activity	16 weeks	1.2 months	Cognition: MoCA CDT Verbal fluency Dual task Depression: GDS	—	No between-group differences for any variables

Baker (2007)	38 powered 63.2	Retirement village >60 years (76.6)	Multicomponent	Group class	Balance 1x/week, aerobic 20 min, 2x/week resistance 3x/week	Wait list control-usual activity	10 weeks	—	Dynamic muscle strength: 1RM Physical performance: 6MWT Static and Dynamic Balance: A progressive test protocols	Physical performance: chair stand stair climb. SPPB Habitual activity: 2MWT PAS for elderly Self-efficacy: Ewert scale Depression: GDS	Greater improvement in EG: hip flexion right (*M* = 20.2), change = 44.1%; left *M* = 21.0, change = 43%; chest press (*M* = 65.0, change = 25.5%) Both groups improved stair climb power and chair stand time No differences in 6MWT

Benavent-Caballer (2016)	51 powered 76.4	Community >65 years (69.0)	Multicomponent (Otago program)	Home exercise with video	Minimum 27 min, 3x/week	Usual activity	16 weeks	—	Physical performance: TUG SPPB	Balance: BBS	Significant improvement in TUG; EG: 7.5^*∗∗*^ ± 2.0 vs CG 8.8 ± 1.9, mean difference −1.3 secs, (95% CI −2.3– −0.1) functional balance; EG: 54.9 ± 2.5 vs CG: 51.4 ± 5.3, mean difference 3.5 ± 9 (5% CI 1.2–5.8)

Bernardelli (2019)	186 underpowered 80	Community ≥75 years (75.6)	Multicomponent + walking encouraged	Physio-led group class	45 min, 1x/week	Wait list control-usual activity	16 weeks	12 months	Physical performance: 400MWT SPPB	Pain: VAS McGill questionnaire Depression: GDS Disability: ODI	All groups improved postintervention, more improvement in EG: 400mwt: mean = −8.4 95% CI−18.6−1.7; SPPB mean = 0.5, 95%CI = 0.1–0.9 No significant differences for other variables

Bernard (2014)	121 convenience sample 100	Community 57–75 years (65.48)	Walking program	Two outdoor supervised walking sessions and one nonsupervised session	40 min. 3x/week	Wait list control-usual activity	24 weeks	—	Depression: BDI	—	Significant improvement in depression compared to CG Mean difference = 2.77 ± 1.12, (95% CI 0.55, −4.99)

Bonura (2014)	98 convenience sample 75.5	Community >65 years (77.0)	Chair yoga and meditation	Group class + home exercise	45 min, 1x/week + 15 min, 6x/week at home	Wait list control-usual activity	6 weeks	1 month	Psychological health: GDS STAXI-2 STAI PGCMS GSES CDSES SCS		Yoga improved more than EG and CG in anger: Cohen's *d* = 0.89, anxiety *d* = 0.27, depression *d* = 0.47, wellbeing *d* = 0.14 and self-efficacy *d* = 0.52

Brown (2009)	154 convenience sample 87.6	Intermediate care and self-care Range 62 to 95 years (79.7)	(1) Multicomponent group exercise (GE) (2) Flexibility and relaxation (FR)	Supervised Supervised	1 hour 2x/week 1 hour 2x/week	Usual activity	24 weeks	—	Cognition Fluid intelligence, working memory, executive functioning Depression GDS PANAS	—	Significantly improved cognition in GE mean = 36.4, significant improvement in PANAS for both EGs and a trend for EGs to reduce depression in those with high GDS scores mean = 5.3 and 8.4

Cassilhas (2007)	62 convenience sample 0	NR (65–75 years∧)	Resistance (1) Moderate intensity (50% of 1 RM) (2) High intensity (80% of 1 RM)	Group class	1 hour 3x/week 1 hour 2x/week	Warm-up and stretching without resistance	24 weeks	—	Cognition Toulouse–Pieron concentration Rey–Osterrieth figure tests WAIS III WSM-R	Depression GDS POMS Quality of life Medical outcomes study SF-36 questionnaire Strength 1RM Body Composition	The moderate intensity EG scored higher than the CG on digit span mean = 0.51, Corsis task mean = 0.97, Rey– Osterrieth immediate recall mean = 8.38, SF-36 mean = −8.94, and POMS depression-dejection mean = −1.89

Choi (2018)	63 powered 93.6	Community >65 years (78 0.2)	Sitting yoga program	Twice a week by direct instruction and twice a week by a peer-volunteer-led videotaped program	30–40 min, 4x/week	Usual activity	12 weeks	—	Sleep quality: PSQI-K	Physical fitness: body composition Flexibility: back stretch test Strength: dynamometer Depression: GDSSF-K	Significant between group differences for grip strength dominant mean = 22.29, SD = ± 6.30 CI = (0.29–3.36). Nondominant mean = 21.13, SD = ±5.49 CI = (0.17–3.35) (0.49e3.69) And other measure of UL strength and flexibility and depression mean = 3.82, SD = ±2.82 CI = (−2.53 to −0.26)

Eyigor (2009)	37 convenience sample 100	Outpatient clinic >65 years (72.35)	Folklore dancing	Group class	60 min, 3x/week	Usual activity	8 weeks	—	Physical performance: TUG SPPB 20 min walk 6 min walk Stair climbing Chair rise Quality of life SF-36 Depression GDS Balance BBS	—	Statistically significant improvement in pre-post for EG in 6MWT mean = 488.8, BBS mean = 55.3 and elements of SF-36: physical function mean = 88.8, general health mean = 77.4, mental health mean = 81.0, and BBS mean = 55.3

Ferraro (2019)	59 powered 60	Community >65 years (74.0)	Inspiratory muscle training	Home exercise unsupervised	2x/day 30 reps	Sham-inspiratory muscle training	8 weeks	—	Physical performance: Sit ups. Biering– Sorensen test Balance: mini-BEST TUG 5x sit to stand. Postural stability	Respiratory function: Spirometry	The EG had significant improvement in TUG mean = 9.2, change = −10.7^*∗∗*^), Sit up (mean = 87.2, change = 45.6) and Mini-BEST mean = 24.1, change = 18.1)

Hackney (2015)	74 underpowered 71.6	Independent living communities ≥55 years (83.2)	Tango dancing	Supervised	90 min 4x/week	Education classes	12 weeks	3 months	Mobility and balance: FSST BBS 6MWT Cadence Body position Cognition: MoCA Trail making	Depression: GDS QoL: SF-12	Significant improvement in EG for mobility: Backward and fast gait speeds mean = 0.59, *n*^2^ = 0.138, mean = 1.2, *n*^2^ = 0.086 and motor-cognitive function, mean = 22.9, *n*^2^ = 0.000. Education improved depression mean = 1.3

Kamegaya (2014)	52 convenience sample 90.3	Community ≥65 years (74.9)	Multicomponent physical and leisure activities	Group class	45 min to 2hours 1x/week	Usual activity	12 weeks	—	Cognition: 5-cognitive test Yamaguchi substitution test WDST	Depression GDS QOL SDL Physical Function: Grip strength TUG 5 m walk time. Functional Reach	Significant improvement on the five-Cog test analogy task, mean = 11.0, and improved QoL mean = 2.0 compared to CG No improvement in physical function or depression

Kamegaya (2012)	30 convenience sample 86.6	Community ≥65 years (74.3)	Physical activity + home exercise + walking program	Group class	45 min 1x/week	Nutrition lecture	12 weeks	—	Cognition: 5-cognitive test WDST	Depression GDS QOL SDL Physical function Grip strength TUG 5 m walk time 1 leg stand	Significant improvement on the five-Cog test, cued recall task mean = 21.0 and WDSST mean = 60.4 (pre-post) No significant change in physical function

Kekalainen (2018)	104 powered 54.8	Community 65–75 years (68.47)	Strength training at 3 different frequencies (RT1, RT2, RT3)	Supervised	1 hour 1–3 months: ALL 2x/week 4–9 months RT1 = 1x/week RT2 = 2x/week RT3 = 3x/week	Usual care	36 weeks	—	Quality of life: WHOQOL-BREF Depression BDI-II	Sence of coherence SOC antonovsky's 13-item scale	Improvement in SoC and environmental QoL in RT2 compared to CG (effect sizes >0.80) compared to RT1 and RT3 (effect sizes >0.50)

Kwok (2011)	40 convenience sample 92.5	Community 66 to 90 years (79.0)	Simplified tai chi	Group class	40 min 1x/week	Towel stretch exercises	8 weeks	—	Cognition: CMMSE CDRS	Physical Function: TUG	The CDRS scores of the CG improved slightly by 2 points. No significant change was found in CMMSE *t* (19) = 0.665, CDRS *t* (19) = −0.891, and TUG *t* (19) = −1.908

Laredo-Aguilera (2018)	38 convenience sample 84.0	Community >65 years (75.8)	Functional exercise training	Group class	60 min 3x/week	Usual activity	10 weeks	—	Depression: GDS Mood: POMS Pain: VAS Sleep: OSQ	—	Significant improvement in pre-posttest for EG in GDS mean = 1.92

Lee (2020)	40 convenience sample 100	Community NR (72.2)	Aquatic exercise	Supervised	60 min 3x/week	NR: assume usual care	12 weeks	—	Mood: POMS	Immune function: Venous samples BMI	Significant improvement within-group analysis in the mood for EG Total scores mean = 22.93

Legault (2011)	73 powered 50.6	Community 70–85 years (76.4)	(1) Multicomponent (2) Multicomponent and cognitive training	Center based sessions and home exercise	150 min 2x/week	health education lectures	16 weeks	2 months 4 months	Cognition: Executive function Self-ordered pointing task 1-Back 2-Back Flanker task Task switching Trail making	Cognition: Memory WMS-III Hopkins verbal learning test	No statistically significant between-group differences in memory

Lustosa (2011)	32 powered 100	Community >65 years (72.0)	Lower limb strength training	Group class	60 min, 3x/week	Wait list - usual activity	10 weeks	—	Physical Performance: TUG 10MWT	Strength: Dynamometry	Statistically significant benefit in pre-posttest for EG in TUG mean = 10.41^*∗∗*^ and gait speed mean = 4.36

Mori (2020)	89 powered 77.5	Community ≥60 years (69.5)	Tai chi	Group class + home exercises	60 min, 1x/week 3 months progress to 2x/week further 9 months	Usual activity	52 weeks	—	Physical performance: TUG Functional reach 10MWT Strength	Arteriosclerosis cardio-ankle vascular index Balance: One leg balance Body weight	Compared with the CG, the EG improved significantly in FRT, GT, and TUG after 6 months mean = 31.7, mean = 4.7, and mean = 4.9 and were maintained after 1 year

Muscari (2010)	120 powered 48	Community NR (69.2)	Endurance (cycle ergometer, treadmill & free-body activity)	Group class	60 min, 3x/week	Lifestyle modification education	52 weeks	—	Cognition: MMSE	Strength: cycle ergometer Power/HR: power/beats	Significant decrease in MMSE score in CG (mean difference −1.21, 95% CI −1.83/−0.60, which differed significantly EG (mean diff = −0.21, 95% CI −0.79/0.37, *p*=0.47

Okumiya (1996)	42 convenience sample 57	Community >75 years (79.0)	Multicomponent	Group class	60 min, 2x/week	Usual activity	24 weeks	—	Cognition: MMSE HDSR VCP-test	Functional performance: TUG Functional reach test Button test	The improvement between groups in TUG mean = 9.9^*∗∗*^, and functional reach mean = 33.1 No significant improvement for MMSE mean = 28.2

Perrig-Chiello (1998)	46convenience sample 39	Community 65 to 95 years old (73.2)	Strengthening	Supervised	1x/week: gym-based machines	NR	8 weeks	12 months	Cognition: DSST: speed, recall, recognition Wellbeing: Self-attentiveness and control belief surveys	Strength: Knee extension power	No significant between group difference for recognition, mean differences = [t (42) = 1.83, *p*=0.07; *t* (43) = 1.77, *p*=0. 08 No significant difference for cognitive speed test (WAIS-NAI). Significant increase in self-attentiveness/self-preoccupation in EG [*t* (22) = 2.83, *p* < 0.01]

Satoh (2014)	89 underpowered 85.3	NR 65–84 years (72.9)	(1) Multicomponent with music (2) Multicomponent without music	Group class	1hour 1x/week 1hour 1x/week	Usual activity	56 weeks	—	Cognition: MMSE RCPM RBMT Word fluency TMT	—	The intragroup analysis, before and after intervention, shows significant improvement in visuospatial assessment EG with music mean = 14.3, compare to CG

Teri (2011)	273 powered 62	Community NR (79.2)	Multicomponent (Seattle protocol) SPA	Group class	60 min, 1x/week (9 classes in 3 months + 5 boosters in 12 months)	Usual activity	12 weeks	6, 12, 18 months	QoL: SF-36 Depression: GDS	Physical performance: 6MWT PACE PAQE	At 3 months, a significantly greater improvement in EG in SF-36 Mean = 2.5 CI 0.4–4.6, and 6MWT Mean = 28.4 CI 6.7–50.1 than CG No significant differences in GDS

Vaughan (2014)	49 underpowered 100	Community 65 to 75 years (68.9)	Multicomponent	Group class	60 min, 2x/week	Wait list control, no formal exercise >60 min/week	16 weeks	12 months	Cognition: COAST Trail making Stroop test Physical function: TUG 6MWT SLST	Brain function: Brain-derived neurotrophic factor	The EG significantly better than the CG in the trail making test A mean = 25.5, CI = −7.8 to −0.6 and test B mean = 53.0, CI = −17.9 to−0.6, Stroop test mean = 101.8, CI = 0.7–9.7, TUG mean *=* 4.9, CI = −2.3 to −1.2, 6MWT mean = 602.0, CI = 73.2–153.1

Vidoni (2015)	101 convenience sample 64	Community ≥65 years (72.9)	Aerobic exercise: Treadmill + other aerobic modalities (3 dose groups)	Supervised	Up to 75 min OR 150 min OR 225 min/week 3-5x/week	Usual activity (sedentary or underactive)	26 weeks	6 months	Physical performance: Physical function test QoL: SF-36 cognition: 16 test cognitive battery		No changes in physical function, perceived function or perceived physical and mental health between groups. Cognitive benefits in low dose EG and visuospatial function higher dose EG in those who adhered to the protocol

Wolfson (1996)	110 convenience sample 41.8	Community ≥75 years (80.0)	(1) Balance, (2) Strength (3) Balance, strength, education (all groups tai chi for 6 months postintervention)	Group class	Group 1 & 2 = 45 min, group 3 = 90 min, 3x/week Tai chi 60 min, 1x/week	Usual activity + education	12 weeks	—	Balance: SOT SLST FBOS Functional performance: Gait velocit*y*	Strength: Lower limb torque	Pre-posttest found significant change for occurrence of LOB in group B mean = 1.4 than CG the B + S and S groups mean = 1.9, mean = 2.1 also showed improvements, but the changes were not significantly different when compared with CG

Yoon (2017)	30 convenience sample NR	Community >65 years (76.3)	(1) Strengthening at low speed (2) Strengthening at high-speed	Supervised	1hour 2x/week	Usual activity + stretching	12 weeks	—	Cognition: MMSE MoCA-K Physical function: SPPB TUG	Strength: Grip and lower limb torque	Significant improvements in MMSE were seen in both the HSPT (*M* = 25.36, change = 20.76 (16.80, 25.82 and LSST (*M* = 24.56, change = 13.91 (5.15, 22.44) groups compared with the CG group. SPPB were increased significantly in the HSPT (*M* = 10.79, change = 32.55 (26.89, 77.39) and LSST (*M* = 10.56, change = 20.27 (8.04, 28.92) groups compared with the CG group

1RM: one-repetition maximum, 400MWT: time to walk 400 meters (seconds), 2MWT: 2-meter walk test, 6MWT: six-minute walk test, 10MWT: 10-meter walk test, BBS: Berg balance scale, BDI: Beck depression inventory, BDI- II: Beck depression inventory II, BMI: body mass index, CDRS: Chinese dementia rating scale, CDSES: chronic disease self-efficacy scales, CDT: clock drawing test, CG: control group, CI: confidence interval, CMMSE: Chinese mini-mental state examination, COAST: cognitive outcome after stroke test, DSST: digit symbol substitution test, EG: exercise group, FBOS: functional base of support, FSST: the four-square step test, GDS: geriatric depression scale, GDSSF-K: geriatric depression scale short form-Korean version, GSES: general self-efficacy scale, HDSR: Hasegawa dementia scale revised, Mini-BEST: balance evaluation system test, MMSE: mini-mental status examination, MoCA: Montreal cognitive assessment, MoCA-K: the Korean version of the Montreal cognitive assessment, NR: not reported, ODI: Oswestry disability index, OSQ: Oviedo sleep questionnaire, PACE: the physician-based assessment and counseling for exercise, PANAS: positive and negative affect schedule, PAS: the physical activity scale, PAQE: the physical activity questionnaire for the elderly, PGCMS: Lawton's PGC morale scale, POMS: profile of mood status, PSQI-K: the Korean version of the Pittsburgh sleep quality index, RCT: randomized controlled trial, RCPM: Raven-colored progressive matrices, RBMT: Rivermead behavioural memory test, SD: standard deviation, SDL: the satisfaction in daily life, SCS: self-control schedule, SF 36: short form health survey, SF-12: the short form health survey, SLST: single leg stance time, SOT: sensory organization test, SPPB: short physical performance battery, STAI: state anxiety inventory, STAXI-2: state anger expression inventory, TMT: trail making test, TUG: timed up and go, VCP-test: visuospatial cognitive performance test, VAS: visual analogue scale, WAIS-III: Weschler adult intelligence scale III, WDST: Weschler digit substitution test, WSM-R: Weschler memory scale-revised, WHOQoL: World Health Organization Quality of Life, WMS-III: wechsler memory scale III. ^*∗∗*^MDC for measure exceeded.

**Table 2:** Description of included studies with frail older adults.

Author (year)	Study design	Sample size Female percentage (%)	Source of participants Age criteria, (mean, range^*∧*^ median ^*∗*^)	Intervention	Mode of delivery	Dose	Comparison	Duration (weeks)	Follow-up (months)	Main outcomes: tool	Other outcomes: tool	Main result
Cardalda (2019)	RCT	77 powered 71.4	Residential care facilities >75 years (84.8)	(1) Strength program with resistance bands (2) Seated calisthenics, mobility, coordination, and games	Group class	60 min 2x/week	Usual recreational and cognitive activities 60 min 2x/week	12 weeks	—	Cognition: MMSE Pfieffer test Physical performance: Barthel index FTSTS	QoL: SF-12	13.4%^*∗∗*^ improvement in MMSE for EG1 with significant difference between all groups. 3.4% improvement in BI in EG1 and 15.9%^*∗∗*^ in the EG2, significant differences between EG2 and other groups Significant differences between EG1 and other groups on QoL for physical component. Improvement of 54.9% for EG1, of 11.9% for EG2 and a deterioration of 6.8% for the CG. In the mental component there were significant differences between EG1, EG2 and the CG group: 31.7% improvement for EG1 and 30.9% improvement for the EG2, CG 9.2% deterioration

Clegg (2014)	RCT	84 convenience sample 71.4	Hospitals, outpatient & GP clinics NR (78.7)	Graded strengthening exercises for mobility	Home based + weekly physio support	<15 min 3x/day 5x/week	Usual care	12 weeks	—	Physical performance: TUG Function: Barthel index	Depression: GDS QoL: EQ-5D	A nonsignificant trend towards a clinically important improved outcome in EG (mean adjusted between-group difference in TUG 28.6, 95% CI −8.5, 65.9). No differences in other outcomes

Conradson (2010)	Cluster-RCT	191 convenience sample 72.7	Residential care facilities 65–100 years (84.75)	High-intensity functional weight-bearing (HIFE program)	Supervised	45 min 5x/2 weeks	OT developed social activity program	12 weeks	3 months	Depression GDS-15 PGCMS	—	No significant differences in GDS or PGCMS between the EG and CG at 3 mth follow-up (GDS mean = 4.35 difference = 0.09) (CI −0.55 to 0.74), PGCMS, mean = 11.52, difference = 0.57 (CI - 0.19 to 1.33) and 6 mth follow-up (GDS, mean = 4.50), difference = 0.25 (CI-0.49 to 0.99) and (PGCMS, mean = 11.56, difference = 0.64) (CI - 0.16 to 1.44)

De Carvalho (2004)	Nonrandomized control trial	37 convenience sample 75.6	Residential care facility 60 to 99 years (NR)	Multicomponent	Group class	60 min 2x/week	Usual activity	24 weeks	—	Depression: GDS Cognition: MMSE	Physical performance: 6MWT Strength (quads) Obstacle course Lower limb function test	Significant improvement in EG in functional tests (OCQTS and OCQLS), lower-limb function, gait velocity, strength, and the GDS, and significantly reduced functional performance OCQLS, lower-limb function, gait velocity, MMSE, and the GDS in CG

Dechamps (2010)	RCT	160 powered 71.8	Residential care facilities >65 years (82.3)	(1) Tai chi (2) Cognition action	Group class	30 min 4x/week 30 to 45 min 2x/week	Usual care	24 weeks	12 months	Function: ADL NPI Cognition: MMSE	Physical performance: TUG Chair rise test. 10MWT Strength (grip) Mood GDS	Significant decline in ADL in CG (adjusted mean difference 1.56; CI 1.02 to 2.10; (*P* < 001)), no significant changes in ADL in EGs, 0.36; CI 0.43 to 1.15 in EG1, and −0.09; CI, −0.90 to 0.72 in EG2 After 6 months, CG significantly worsened TUG, CI= (−1.7 to 3.4) GDS improved at 6 months in all 3 groups

Diegelmann (2018)	Nonrandomized control trial	163 convenience sample 71.7	Residential care facilities 53–100 years(83.5) ^*∗*^	4 different group class options with multicomponent exercise	Group class	Minimum 45 min 2x/week	Wait list-usual activity	12 weeks	3 months	Depression: GDS	—	Depressive symptoms increased in the CG (*β* = 0.03, *p* < 0.05). This was different for the EG who showed significantly less depressive symptoms than the CG at study end and follow-up (*β* = −0.22 and *β* = −0.27, both *p* < 0.05)

Dorner (2007)	RCT	30 convenience sample 76.6	Residential care facility >75 years (86.8)	Strength & balance training	Group class	50 min 3x/week	NR -usual activity?	10 weeks	—	Cognition: MMSE Depression: GDS	Function: FIM Tinetti Barthel index Strength BMI	No significant difference between CG and EG on MMSE (*p*=0.1), ANOVA-RM test mean = 23.9. Significant difference in mean strength (*p* < 0.001) ANOVA-RM test) mean = 4.44 No significant difference in lean body mass mean = 44.2, Tinetti score mean = 11.1, BI mean = 64.7, FIM mean = 81.5 or GDS mean = 1.47

Halvarsson (2011)	RCT	59 convenience sample 71.1	Community ≥65 years (77.0)	Multi-level balance training (i.e., dual, or multi-cognitive and/or motor tasks), individually adjusted	Group class with physio	45 min 3x/week	Wait list -usual activities	12 weeks	—	Balance: Fear of falling: FES-I step-execution time	Gait: GAITRite Depression GDS	Significant improvement in the FES-I (*P*=0.008), in step-execution mean = 1.73 and cadence at normal speed mean = 113 and, velocity at fast speed, mean = 1.60 No significant group differences in depression

Hauer (2003)	RCT	57 convenience sample 100	Outpatient geriatric hospital >75 years (84.3)	Multicomponent	Group class with physio	45 min 3x/week + physio 25 min, 2x/week	Placebo activities 60 min, 3x/week + physio 25 min, 2x/week	12 weeks	3 months 24 months	Physical performance: TUG Tinetti's POMA Gait speed (m/s), functional reach test IADL Fear of falling Strength Chair rise Physical activity	Depression: GDS-SF PGCMS	Significantly improved TUG in EG mean = 19.7^*∗∗*^, differences between the groups were significant for TUG at 2 years follow-up mean = 25.5^*∗∗*^ No significant differences in emotional status between groups

Hsu (2016a)	RCT	60 powered 63.3	Residential care facility >65 years (81.25)	Seated tai chi	Group class	40 min 3x/week	Usual exercise and recreational activities	26 weeks	—	Mood: POMS-SF Self-efficacy: SEE	—	Significantly lower mood in EG on POMS-SF (mean 3.56 SD 3.71) than the CG (mean 7.16 SD 6.36) (*F* [1, 58] = 7.15; *p* < 0.05). EG significantly higher SEE (mean, 35.66 SD 36.83) than CG (mean, SD 15.30–26.43) (*F* [1, 58] = 6.05; *p* < 0.05)

Hsu (2016b)	RCT	60 powered 63.3	Residential care facility ≥65 years (81.2)	Seated tai chi	Group class	40 min 3x/week	Usual exercise and recreational activities	26 weeks	—	QoL: WHOQoL Depression: GDS-SF	—	Significantly lower GDS-SF scores in EG (mean = 3.76, SD = 3.65) than CG (mean = 7.76, SD = 5.15) effect size = 0.89. Significantly higher QOL in EG (mean = 3.47, SD = 0.57) effect size 0.56

Langlois (2013)	RCT	72 convenience sample 77.7	Community NR (61 to 89∧)	Aerobic & strength exercises	Group class	60 min 3x/week	Wait list: usual activity	12 weeks		Cognition: MMSE Memory, executive function, and verbal reasoning QoL: QLSI	Physical performance: 6MWT TUG Gait speed (s)	Significant between group difference in 6-MWT *F* (1, 68) = 4.79, *p*=0.03), cognitive performance *F* (1, 68) = 4.45, *p*=0.039, and QoL, *F* (1, 68) = 3.97, *p*=0.05

MacRae (1996)	Nonrandomized control trial	31 Convenience sample 96.7	Residential care facilities NR (90.5)	Supervised walking program at a self-selected pace	Walk with research assistant	Up to 30 min 5x/week	Wait list control social visit 30 min. 5x/week for 12 weeks, then 10 weeks walk program	12 weeks then a further 10 weeks	5 months	QoL: COOP Depression: GDS	Physical performance: TUG Walk time/distance Balance: Tinetti	Significant improvement in EG in max. Walk endurance time by 77% and distance by 92%, no significant change in walk speed. No significant change in these variables in CG. No significant changes for physical activity, mobility, and quality of life for EG or CG

Netz (1994)	Block design RCT	31 convenience sample 51.6	Hospital inpatients, geriatric and psychiatric ward NR (71.0)	Multicomponent	Group class	45 min 3x/week	Supervised social activity. 45 min 3x/week	8 weeks	—	Cognition: MMSE Depression: GDS	—	No significant between group differences for MMSE Both groups had a significant improvement on GDS (*p* < 0002) greatest improvement in EG

Sattin (2005)	Cluster RCT	311 powered 94.4	Community >75 years (70 to 97∧)	Tai chi	Group class	60 to 90 min, 2x/week	Wellness education program + handout 60 min, 1x/week	52 weeks	—	Physical performance: Functional reach 10MWT Level of activity	Depression CES-D Falls: Fear of falling Fall frequency ABC & FE*S*	Significantly improved ABC in EG at 8 months (57.9 vs. 49.0, *P* < .001) and at 12 months (59.2 vs. 47.9, *P* < .001). Mean FES significantly improved in EG at 8 months (18.4 vs. 20.5, *P* = 0.01) and 12 months (17.6 vs 21.2, *P* < .001)

Sink (2015)	RCT	1476 powered 67.6	Community NR (70 to 89∧)	Multicomponent	Group class + home exercises	50 min, 1x/week + home exercise 3-4x/week	Health & exercise education 60 to 90 min 1x/week	104 weeks	—	Cognition: WAIS - digit symbol coding task Hopkins verbal learning test Digital word recall Count back task Task switching Flanker task	—	No significant group difference for the symbol coding task (mean difference, −0.01 points [95% CI, −0.80 to 0.77 points], *p*=0.97) or Hopkins task (mean difference, −0.03 words [95% CI, −0.29 to 0.24 words], *p*=0.84)

Timonen (2002)	RCT	68 convenience sample 100	Geriatric ward of hospital >75 years (83.0)	Resistance & functional exercises	Group class	90 min incl. warmup, 2x/week	Unsupervised home program	10 weeks	3 months 9 months	Depression: Zung self-rating depression scale	—	Significant improvement in mood in EG compared to CG: mean 3.1 (SD 9.0) vs. 1.3 (SD 7.6) (*p*=0.048) and maintained at 3-month follow-up −2.6 (SD 7.7) points vs. +3.5 (SD 9.7) points (*p*=0.01)

Topp (2005)	RCT	131 convenience sample 71.7	Community ≥65 years (73.2 to 75.9∧)	(1) Strength training (2) Aerobic walking (3) Combined resistance and aerobic	Group class + home exercises	45 min 1x/week + home exercise 2x/week	Usual activity	16 weeks	—	Physical performance: Arm curl. Sit-to-stand. Down/up off the floor Stair ascent/descent		All EGs significantly higher (15% to 21%) than CG with most consistent gains over the six measures in EG3

Tsugawa 2020	nonrandomized RCT	37n convenience sample 59.4	Residential care facility NR (84.5)	Multicomponent	Supervised	40 min 2×/week	Usual activity	56 weeks	—	Cognition: MMSE Trail making Physical Performance: Barthel Index	Depression: GDS muscle mass: Skeletal mass index Strength: Grip	EG significantly improved in MMSE (*p*=0.037) and grip force (*p*=0.007) compared to CG. There were no significant changes in GDS (*p*=0.244) or BI (*p*=0.186) and trail making (*p*=0.242) in either group

vandeRest (2014)	RCT	127 powered 60.6	Community >65 years (69.0)	Strength training	Supervised 1 : 1	3 to 4 sets on six machines 2x/week	Usual activity	24 weeks	—	Cognition: Neuropsychological test battery	—	CG improved compared to the EG on verbal fluency (mean 2.4 ± 4.1 compared to mean −0.6 ± 4.1 respectively, (*p* < 0.01). Processing speed improved significantly in the EG (mean change = 0.08 ± 0.51) compared to CG (mean change = −0.23 ± 0.19) (*p* = 0.04)

Varela (2018)	RCT	39 convenience sample 76.9	Residential care facility >65 years (80.7)	Cycling on recumbent bike at self-selected intensity	Supervised 1 : 1 with physio	Minimum 15 min each day	Recreational activities	68 weeks	—	Cognition: MMSE Fuld object memory evaluation SDMT physical performance: TUG and Katz index WHO Questionnaire	—	Significant improvements were observed in the exercise group for global cognition and attention, visual scanning, and processing speed

Vedovelli (2017)	RCT	29 convenience sample 100	Residential care facility ≥75 years (80.1)	Strength training with resistance bands + progressive walking	Group class	60 min up to 30 min, 3x/week	Usual activity	12 weeks	—	Depression: BDI Anxiety: BAI Cognition: Digit span Stroop Trail making Contextual memory Forward and backward digit span tests of the Wechsler adult intelligence scale	Physical performance: 6MWT and 30-s chair stand test Strength Aerobic conditioning	Improvement in strength [*F* (2,38) = 43.387, *η*2*ρ* = 0.695, *p* < 0.001], aerobic conditioning [*F* (2, 38) = 17.975, *η*2*ρ* = 0.486, *p* < 0.001], and depression [*F* (2,38) = 9.09, ƞ2 = 0.324, *p* = 0.001], forward digit span [*F* (2,38) = 8.665, *η*2*ρ* = 0.95, *p* < 0.001], backward digit span [*F* (2,38) = 3.994, *η*2*ρ* = 0.174, *p* = 0.027], trail making B [*F* (2,38) = 6.627, *η*2*ρ* = 0.241, *p* = 0.037], and Stroop [*F* (2,38) = 8.332, *η*2*ρ* = 0.149, *p* = 0.002] tasks in EG

Venturelli (2010)	RCT	30 convenience sample 100	Residential care facility ≥65 years (83.7)	Strength training	Group class	45 min 3x/week	Usual activity	12 weeks	—	Cognition: MMSE Physical Performance: 1RM arm curl Back scratch Arm circumference. Barthel index	—	EG maintained MMSE after the training period (mean = 23.0 ± 1.4), but CG showed a significant decrease (mean = 17.5 ± 2.1) Significant improvement in the BI for EG (mean = 34.8 ± 14.9), whereas CG maintained a similar score (19.3 ± 11.9)

Williamson (2009)	RCT	102 powered 70.6	NR 70 to 89 years (77.1)	Multicomponent	Group class	40 to 60 min 3x/week	Health education in group class weekly and then monthly for the final 6-months	52 weeks	—	Cognition: DSST Rey Stroop Modified mini-mental state examination Physical performance: SPPB	—	Group differences were not significant, but improvements in cognitive scores were associated with improvements in physical function. DSST significantly correlated with change in SPPB (*r* = 0.38, *p* = 0.0002), in the chair stand score (*r* = 0.26, *p* = 0.012), in the balance score (*r* = 0.21, *p* = 0.046), and in 400-m gait speed (*r* = 0.15, *p* = 0.147)

Wolf (2001)	RCT	77 underpowered 72.7	Residential care facilities >75 years (84.0)	Balance training	Group class	30 min 2or 3x/ week	Recreational activities	4 to 6 weeks	1 month 12 months	Balance: BBS DGI Mood: HADS	Fear of falling: VAS	EG improved significantly more on the BBS and DGI than the CG (*p* ≤ 0.001, *p* ≤ 0.001). No significant change for other variables

Wolf (2003)	RCT	200 powered 80.6	Community >70 years (76.2)	(1) Tai chi: balance focus (2) Computerized balance training	Home exercise	15 min per day 2xweek	Education sessions 60 min 1x/week	15 weeks	4months	Physical performance: IADL Strength Flexibility Fear of falling Health perception Endurance	Mood: CES-D Sleep Quality Falls frequency	Grip strength declined in all groups. Also observed for distance ambulated over 12 minutes. Interestingly, the BT and ED groups increased their walking distance (0.01 mile), whereas the TC group reduced the distance travelled by 0.02 mile (Tukey pairwise comparison, *p* = 0.040). Fear of falling and intrusiveness reduced in EG1 compared with CG (*p* = 0.046 and *p* = 0.058, respectively)

Yoon (2018)	RCT	43 convenience sample 69.7	Community NR (73.94)	A high-speed strength training program	Supervised	1 hour 3×/week	Usual activities + stretching (using elastic band) + balance	16 weeks	—	Cognition: Memory Rey-15 FAB Processing speed Cognitive flexibility Working memory Physical performance: SPPB TUG Strength Gait speed. Frailty score	—	Significantly improved performance for cognitive function (processing speed (mean = 48.26, effect size = 0.21), frontal assessment battery (mean = 13.70, effect size = 0.74) physical function (TUG mean = 9.26^*∗∗*^, effect size = 0.65 in EG between group differences

∧: Age range reported instead of mean age,  ^*∗*^: Median, 1RM: 1 repetition maximum 6MWT: 6-minute walk test, 10MWT: 10 meter walk test, ABC: activity-specific balance confidence scale, ADL: activities of daily living, BAI: beck anxiety inventory, BBS: berg balance scale, BDI: Beck depression inventory, BMI: body mass index, CES-D: center for epidemiological studies-depression, CG: control group, CI: confidence interval, COOP: dartmouth cooperative functional assessment charts, DGI: dynamic gait index, DSST: digital symbol substitution test, EG: exercise group EQ-5D: EuroQoL 5-dimension instrument, FAB: frontal assessment battery, FES-I: falls efficacy scale international, FES: the falls efficacy scale, FIM: functional inventory measure, FTSTS: five timed sit to stand, GDS: geriatric depression scale, GDS-SF: the geriatric depression scale-short form (Chinese version), GP: general practitioners, HADS: hospital anxiety and depression scale, HSPT: high-speed power training, IADL: instrumental activities of daily living, MMSE: mini-mental scale examination, M/S: meters per second, NPI: neuro-psychiatric inventory, NR: not reported, OCQTS: obstacle course quantitative score, OCQLS: obstacle course qualitative score, PGCMS: Philadelphia geriatric center morale scale, POMS-SF: profile of mood states short form, POMA: performance oriented mobility assessment, QLSI: quality of life systemic inventory questionnaire, QoL: quality of life, RCT: randomized controlled trial, SD: standard deviation, SDMT: symbol digit modalities test, SEE: self-efficacy for exercise, SF-12: the health questionnaire 12-item short form survey, SPPB: short physical performance battery, TUG: timed-up-and-go, VAS: visual analogue scale, WAIS: Wechsler adult intelligence scale, WHO: World Health Organization, WHOQoL: World Health Organization Quality of Life. ^*∗∗*^MCD for measure exceeded.

**Table 3:** Summary of results: number of studies in each category with references.

Exercise type	Outcome measure category	Population		Significant improvement (vs. control group) postintervention		Studies that reported follow-up^a^		Significant improvements reported at follow^b^	
		Healthy older adult	Frail older adult	Healthy older adult	Frail older adult	Healthy older adult	Frail older adult	Healthy older adult	Frail older adult
Multicomponent exercise	Physical health and function	*n* = 10 [22, 35, 38–40, 42, 44, 45, 48, 50]	*n* = 9 [28, 31, 59, 60, 65, 68–71]	*n* = 7 [22, 35, 38, 42, 45, 48, 50]	*n* = 6 [28, 31, 59, 60, 68, 71]	*n* = 2 [22, 48]	*n* = 1 [71]	*n* = 2 [22, 48]	*n* = 1 [71]
	Cognitive function	*n* = 8 [38–41, 43, 46–48]	*n* = 8 [28, 29, 30, 59, 60, 65, 68, 72]	*n* = 5 [39–41, 47, 48]	*n* = 4 [28, 29, 65, 68]	*n* = 1 [48]	N/A	*n* = 1 [48]	N/A
	Mental health and wellbeing	*n* = 9 [22, 35, 39, 40, 45, 46, 47, 48, 50]	*n* = 8 [59, 60, 65, 67, 68, 70–72]	*n* = 4 [35, 45, 47, 49]	*n* = 3 [59, 67, 72]	N/A	*n* = 2 [67, 72]	N/A	*n* = 2 [67, 72]
	Quality of life	*n* = 3 [22, 39, 40]	*n* = 1 [28]	*n* = 2 [22, 39]	*n* = 1 [28]	*n* = 1 [22]	N/A	*n* = 1 [22]	N/A
	Activity of daily living	N/A	*n* = 4 [60, 68, 70, 71]	N/A	*n* = 1 [71]	N/A	*n* = 1 [71]	N/A	*n* = 1 [71]

Tai chi exercise	Physical health and function	*n* = 2 [37, 55]	*n* = 3 [25, 34, 57]	*n* = 1 [37]	*n* = 3 [25, 34, 57]	N/A	*n* = 1 [34]	N/A	N/A
	Cognitive function	*n* = 1 [55]	N/A	*n* = 1 [55]	N/A	N/A	N/A	N/A	N/A
	Mental health and wellbeing	N/A	*n* = 4 [25, 34, 57, 61]	N/A	*n* = 1 [61]	N/A	N/A	N/A	N/A
	Quality of life	N/A	*n* = 1 [61]	N/A	*n* = 1 [61]	N/A	N/A	N/A	N/A
	Activity of daily living	N/A	*n* = 2 [25, 34]	N/A	*n* = 1 [25]	N/A	*n* = 1 [25]	N/A	N/A

Strength exercises	Physical health and function	*n* = 4 [42, 51–53]	*n* = 4 [31, 58, 63, 66]	*n* = 4 [42, 51–53]	*n* = 4 [31, 58, 63, 66]	*n* = 1 [53]	N/A	*n* = 1 [53]	N/A
	Cognitive function	*n* = 4 [18, 46, 51, 53]	*n* = 4 [32, 58, 63, 66]	*n* = 3 [18, 51, 53]	*n* = 4 [32, 58, 63, 66]	*n* = 1 [53]	N/A	*n* = 1 [53]	N/A
	Mental health and wellbeing	*n* = 4 [18, 46, 53, 54]	N/A	*n* = 1 [18]	N/A	N/A	N/A	N/A	N/A
	Quality of life	*n* = 3 [18, 53, 54]	*n* = 1 [63]	*n* = 2 [18, 54]	*n* = 1 [63]	N/A	N/A	N/A	N/A
	Activity of daily living	N/A	*n* = 2 [63, 66]	N/A	*n* = 2 [63, 66]	N/A	N/A	N/A	N/A

Aerobic exercises	Physical health and function	*n* = 2 [23]	*n* = 3 [31, 62, 64]	N/A	*n* = 2 [31, 62]	N/A	N/A	N/A	N/A
	Cognitive function	*n* = 2 [21, 23]	*n* = 1 [64]	*n* = 2 [21, 23]	N/A	N/A	N/A	N/A	N/A
	Mental health and wellbeing	*n* = 1 [16]	*n* = 2 [24, 62]	*n* = 1 [16]	N/A	N/A	N/A	N/A	N/A
	Quality of life	*n* = 1 [23]	*n* = 1 [62]	N/A	N/A	N/A	N/A	N/A	N/A

Dancing: Turkish folklore or tango	Physical health and function	*n* = 2 [36, 56]	N/A	*n* = 2 [36, 56]	N/A	*n* = 1 [56]	N/A	*n* = 1 [56]	N/A
	Cognitive function	*n* = 1 [56]	N/A	*n* = 1 [56]	N/A	*n* = 1 [56]	N/A	*n* = 1 [56]	N/A
	Mental health and wellbeing	*n* = 2 [36, 56]	N/A	*n* = 1 [56]	N/A	*n* = 1 [56]	N/A	*n* = 1 [56]	N/A
	Quality of life	*n* = 1 [36]	N/A	*n* = 1 [36]	N/A	N/A	N/A	N/A	N/A

Balance exercise	Physical health and function	*n* = 1 [42]	*n* = 3 [26, 33, 34]	*n* = 1 [42]	*n* = 3 [26, 33, 34]	N/A	*n* = 1 [33]	N/A	N/A
	Mental health and wellbeing	N/A	*n* = 3 [26, 33, 34]	N/A	N/A	N/A	N/A	N/A	N/A

Yoga	Mental health and wellbeing	*n* = 1 [17]	N/A	*n* = 1 [22]	N/A	*n* = 1 [22]	N/A	*n* = 1 [22]	N/A

Inspiratory training	Physical health and function	*n* = 1 [19]	N/A	*n* = 1 [19]	N/A	N/A	N/A	N/A	N/A

Aquatic exercise	Mental health and wellbeing	*n* = 1 [20]	N/A	*n* = 1 [20]	N/A	N/A	N/A	N/A	N/A

Flexibility and relaxation	Cognitive function	*n* = 1 [47]	N/A	N/A	N/A	N/A	N/A	N/A	N/A
	Mental health and wellbeing	*n* = 1 [47]	N/A	*n* = 1 [47]	N/A	N/A	N/A	N/A	N/A

^a^follow-up data collection varied from 1 month to 24 months; ^b^significant improvement was reported at the first follow-up for each study.

## Data Availability

All data used in this systematic review had been published in original articles from which it was extracted. In some cases, conversions and calculations were conducted to create homogeneity of the data. All data used in meta-analysis will be stored for years in accordance with ethics guidelines and is available on request.

## Additional Points

*Protocol Registration*. PROSPERO (CRD42020173465, on 28 April 2020).

## Supplementary Materials

Supplementary Materials Sapp 1: Example search engine terms as used in MEDLINE. Sapp 2: Quality assessment of the included studies using the PEDRO scale. Sfig 3: The effect of tai chi on specific outcomes. Sfig 4: The effect of strength training on specific outcomes. Sfig 5: The effect of aerobic exercise on the physical health and function of frail older adults. Sfig 6: The effect of dancing on physical health and function of healthy older adults. Sfig 7: Visual representation of the meta-analysis findings.
