# Supplementary material for: Physical Exercise for Healthy Older Adults and Those with Frailty: What Exercise Is Best and Is There a Difference? A Systematic Review and Meta-Analyses
Source: Curr Gerontol Geriatr Res. 2024 Jul 5;2024:5639004. doi: 10.1155/2024/5639004 (PMC11458270; doi:10.1155/2024/5639004)
Supplement: Supplementary Materials — Sapp 1: Example search engine terms as used in MEDLINE. Sapp 2: Quality assessment of the included studies using the PEDRO scale. Sfig 3: The effect of tai chi on specific outcomes. Sfig 4: The effect of strength training on specific outcomes. Sfig 5: The effect of aerobic exercise on the physical health and function of frail older adults. Sfig 6: The effect of dancing on physical health and function of healthy older adults. Sfig 7: Visual representation of the meta-analysis findings. [file 5639004.f1.zip › Appendix 2.docx]

**Table 1: Quality assessment of the included studies using PEDRO scale**

1. **Health older adults**

| HEALTHY | Criteria Item | | | | | | | | | | | |
| --- | --- | --- | --- | --- | --- | --- | --- | --- | --- | --- | --- | --- |
| Study | 1 | 2 | 3 | 4 | 5 | 6 | 7 | 8 | 9 | 10 | 11 | total /10 |
| Ansai et al 2015 | YES | 1 | 1 | 1 | 0 | 0 | 0 | 1 | 1 | 1 | 1 | 7 |
| Baker et al 2007 | YES | 1 | 1 | 1 | 0 | 0 | 1 | 1 | 1 | 1 | 1 | 8 |
| Benavent-Caballer et al 2016 | YES | 1 | 1 | 1 | 0 | 0 | 1 | 0 | 1 | 1 | 1 | 7 |
| Bernardelli  et al 2018 | YES | 1 | 0 | 1 | 0 | 0 | 0 | 0 | 1 | 1 | 1 | 5 |
| Bernard et al 2014 | YES | 1 | 0 | 1 | 0 | 0 | 0 | 1 | 1 | 1 | 1 | 6 |
| Bonura et al 2014 | YES | 1 | 0 | 1 | 0 | 0 | 0 | 0 | 0 | 1 | 0 | 3 |
| Brown et al 2009 | YES | 1 | 0 | 1 | 0 | 0 | 0 | 0 | 1 | 1 | 1 | 5 |
| Cassilhas  et al 2007 | NO | 1 | 0 | 1 | 0 | 0 | 0 | 1 | 0 | 1 | 1 | 5 |
| Choi et al. 2018 | YES | 1 | 0 | 1 | 0 | 0 | 0 | 0 | 1 | 1 | 1 | 5 |
| Eyigor et al 2009 | YES | 1 | 0 | 1 | 0 | 0 | 1 | 1 | 0 | 1 | 1 | 6 |
| Ferraro et al 2019 | YES | 1 | 0 | 1 | 1 | 0 | 1 | 0 | 0 | 1 | 1 | 6 |
| Hackney et al 2015 | YES | 0 | 0 | 0 | 0 | 0 | 1 | 1 | 1 | 1 | 1 | 5 |
| Kamegaya  et al 2014 | YES | 1 | 0 | 1 | 0 | 0 | 0 | 1 | 0 | 1 | 1 | 5 |
| Kamegaya  et al 2012 | YES | 1 | 0 | 1 | 0 | 0 | 0 | 0 | 1 | 1 | 1 | 5 |
| Kekalainen  et al 2018 | YES | 1 | 0 | 1 | 0 | 0 | 0 | 1 | 1 | 1 | 1 | 6 |
| Kwok et al 2011 | NO | 0 | 1 | 1 | 0 | 0 | 0 | 1 | 0 | 1 | 1 | 5 |
| Laredo-Aguilera et al 2018 | YES | 1 | 0 | 1 | 0 | 0 | 0 | 1 | 0 | 1 | 1 | 5 |
| Lee et al 2020 | YES | 0 | 0 | 1 | 0 | 0 | 0 | 0 | 0 | 1 | 1 | 3 |
| Legault et al 2011 | YES | 1 | 0 | 1 | 0 | 0 | 0 | 1 | 1 | 1 | 1 | 6 |
| Lustosa et al 2011 | YES | 1 | 0 | 1 | 0 | 0 | 1 | 1 | 0 | 1 | 1 | 6 |
| Mori et al 2020 | YES | 0 | 0 | 1 | 0 | 0 | 0 | 0 | 0 | 1 | 1 | 3 |
| Muscari et al 2010 | YES | 1 | 0 | 1 | 0 | 0 | 1 | 1 | 1 | 1 | 1 | 7 |
| Okumiya et al 1996 | YES | 1 | 0 | 1 | 0 | 0 | 1 | 1 | 0 | 1 | 1 | 6 |
| Perrig-Chiello et al 1998 | NO | 1 | 0 | 1 | 0 | 0 | 0 | 1 | 0 | 1 | 1 | 5 |
| Satoh et al 2014 | YES | 0 | 1 | 0 | 0 | 0 | 0 | 0 | 0 | 1 | 1 | 3 |
| Teri et al 2011 | YES | 1 | 0 | 0 | 0 | 0 | 0 | 1 | 1 | 1 | 1 | 5 |
| Vaughan et al 2014 | YES | 1 | 1 | 1 | 0 | 0 | 1 | 1 | 1 | 1 | 1 | 8 |
| Vidoni et al 2015 | YES | 1 | 1 | 1 | 0 | 0 | 1 | 0 | 1 | 1 | 1 | 7 |
| Wolfson et al 1996 | YES | 1 | 0 | 1 | 0 | 0 | 1 | 1 | 0 | 1 | 1 | 6 |
| Yoon et al 2017 | YES | 1 | 0 | 1 | 0 | 0 | 0 | 0 | 0 | 1 | 1 | 4 |
| Total /30 | 27 | 25 | 7 | 27 | 1 | 0 | 11 | 18 | 15 | 30 | 29 | 163 |

**b) Frail older adults**

| FRAIL | Criteria Item | | | | | | | | | |  |  |
| --- | --- | --- | --- | --- | --- | --- | --- | --- | --- | --- | --- | --- |
| Study | 1 | 2 | 3 | 4 | 5 | 6 | 7 | 8 | 9 | 10 | 11 | total /10 |
| Clegg et al 2014 | YES | 1 | 1 | 1 | 0 | 0 | 1 | 0 | 1 | 1 | 1 | 7 |
| Conradsson et al 2010 | YES | 1 | 1 | 1 | 0 | 0 | 1 | 1 | 1 | 1 | 1 | 8 |
| de Carvalho Bastone  et al 2004 | YES | 0 | 0 | 1 | 0 | 0 | 0 | 1 | 0 | 1 | 1 | 4 |
| Dechamps et al 2010 | YES | 1 | 1 | 1 | 0 | 0 | 0 | 1 | 1 | 1 | 1 | 7 |
| Diegelmann et al 2018 | YES | 0 | 0 | 1 | 0 | 0 | 0 | 1 | 1 | 1 | 1 | 5 |
| Dorner et al 2007 | YES | 1 | 0 | 1 | 0 | 0 | 1 | 0 | 0 | 1 | 1 | 5 |
| Halvarsson et al 2011 | YES | 1 | 1 | 1 | 0 | 0 | 1 | 1 | 0 | 1 | 1 | 7 |
| Hauer et al 2003 | YES | 1 | 0 | 1 | 0 | 0 | 1 | 0 | 0 | 1 | 1 | 5 |
| Hsu et al 2016a | YES | 1 | 0 | 1 | 0 | 0 | 0 | 1 | 1 | 1 | 1 | 6 |
| Hsu et al 2016b | YES | 1 | 0 | 1 | 0 | 0 | 0 | 1 | 1 | 1 | 1 | 6 |
| Langlois  et al 2013 | YES | 1 | 0 | 0 | 0 | 0 | 0 | 1 | 1 | 1 | 1 | 5 |
| MacRae  et al 1996 | YES | 0 | 0 | 1 | 0 | 0 | 0 | 1 | 1 | 0 | 1 | 4 |
| Cardalda  et al 2019 | YES | 1 | 0 | 1 | 0 | 0 | 0 | 1 | 1 | 1 | 1 | 6 |
| Netz et al 1994 | YES | 1 | 0 | 0 | 0 | 0 | 1 | 0 | 0 | 1 | 0 | 3 |
| Sattin et al 2005 | YES | 1 | 0 | 1 | 0 | 0 | 1 | 0 | 1 | 1 | 1 | 6 |
| Sink et al 2015 | YES | 1 | 0 | 1 | 0 | 0 | 1 | 1 | 1 | 1 | 1 | 7 |
| Timonen  et al 2002 | YES | 1 | 1 | 0 | 0 | 0 | 0 | 0 | 0 | 1 | 1 | 4 |
| Topp et al 2005 | YES | 1 | 0 | 1 | 0 | 0 | 0 | 1 | 1 | 1 | 1 | 6 |
| Tsugawa  et al 2020 | YES | 0 | 0 | 1 | 0 | 0 | 0 | 1 | 1 | 1 | 1 | 5 |
| van de Rest et al 2014 | YES | 1 | 1 | 1 | 1 | 0 | 0 | 1 | 1 | 1 | 1 | 8 |
| Varela et al 2018 | YES | 1 | 0 | 1 | 0 | 0 | 1 | 0 | 1 | 1 | 1 | 6 |
| Vedovelli  et al 2017 | YES | 0 | 0 | 1 | 0 | 0 | 0 | 1 | 0 | 1 | 1 | 4 |
| Venturelli et al 2010 | YES | 1 | 0 | 1 | 0 | 0 | 1 | 0 | 0 | 1 | 1 | 5 |
| Williamson et al 2009 | YES | 1 | 0 | 1 | 0 | 0 | 1 | 1 | 0 | 1 | 1 | 6 |
| Wolf et al 2001 | YES | 1 | 1 | 1 | 0 | 0 | 1 | 1 | 1 | 1 | 1 | 8 |
| Wolf et al 2003 | YES | 1 | 0 | 1 | 0 | 0 | 0 | 1 | 0 | 1 | 1 | 5 |
| Yoon et al 2018 | NO | 1 | 0 | 1 | 0 | 0 | 0 | 0 | 0 | 1 | 1 | 4 |
| Total / 27 | 26 | 22 | 7 | 24 | 1 | 0 | 12 | 18 | 16 | 26 | 26 | 152 |

Table legend: Yes = 1; No = 0; Item 1 eligibility criteria does not contribute to total score.

Criteria Items: 1. Eligibility criteria specified 2. Random allocation 3. Concealed allocation 4. Baseline similarity 5. Participant blinding 6. Therapist blinding 7. Assessor Blinding 8. Outcomes measured 9. Intervention provided 10. Between group comparisons 11. Outcome reported.
