# Supplementary material for: Physical Exercise for Healthy Older Adults and Those with Frailty: What Exercise Is Best and Is There a Difference? A Systematic Review and Meta-Analyses
Source: Curr Gerontol Geriatr Res. 2024 Jul 5;2024:5639004. doi: 10.1155/2024/5639004 (PMC11458270; doi:10.1155/2024/5639004)
Supplement: Supplementary Materials — Sapp 1: Example search engine terms as used in MEDLINE. Sapp 2: Quality assessment of the included studies using the PEDRO scale. Sfig 3: The effect of tai chi on specific outcomes. Sfig 4: The effect of strength training on specific outcomes. Sfig 5: The effect of aerobic exercise on the physical health and function of frail older adults. Sfig 6: The effect of dancing on physical health and function of healthy older adults. Sfig 7: Visual representation of the meta-analysis findings. [file 5639004.f1.zip › Figure 7 Visual representation of the meta-analysis findings (2).docx]

**Figure 7: Visual representation of the meta-analysis findings**

Mobility

Mental health

Activity of Daily Living

Mobility

Mobility

Activity of Daily Living

Quality of Life

Mental health

Cognitive function

Mental health

Physical function

Mental health

Physical function

Physical function (TUG)

Cognitive function

Physical function (TUG)

Healthy

Frail

Healthy

Healthy

Frail

Physical function

Cognitive function

Cognitive function

Frail

**Multicomponent exercise**

**Tai chi exercise**

**Strength exercise**

Frail

Physical function

Healthy

**Aerobic exercise**

**Dancing**
