# Supplementary figures and images for: Physical Exercise for Healthy Older Adults and Those with Frailty: What Exercise Is Best and Is There a Difference? A Systematic Review and Meta-Analyses
Source: Curr Gerontol Geriatr Res. 2024 Jul 5;2024:5639004. doi: 10.1155/2024/5639004 (PMC11458270; doi:10.1155/2024/5639004)

**Figure** **6: The effect of dancing on physical health and function of healthy older adults**


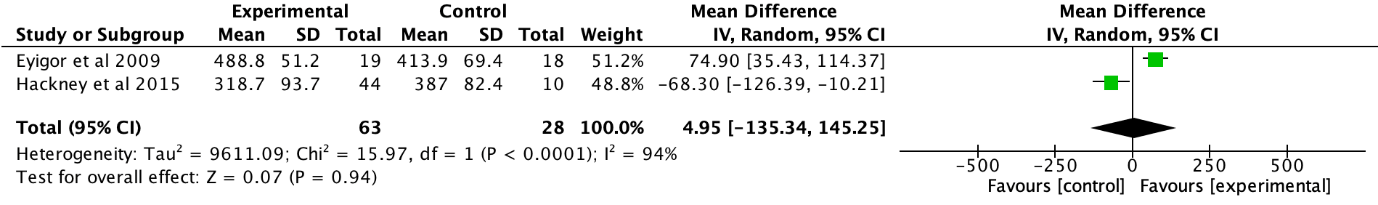

Supplement: Supplementary Materials — Sapp 1: Example search engine terms as used in MEDLINE. Sapp 2: Quality assessment of the included studies using the PEDRO scale. Sfig 3: The effect of tai chi on specific outcomes. Sfig 4: The effect of strength training on specific outcomes. Sfig 5: The effect of aerobic exercise on the physical health and function of frail older adults. Sfig 6: The effect of dancing on physical health and function of healthy older adults. Sfig 7: Visual representation of the meta-analysis findings. [file 5639004.f1.zip › Figure 6 The effect of dancing on physical health and function of healthy older adults (2).docx]
